# A survey on the awareness, current management, and barriers for non-alcoholic fatty liver disease among the general Korean population

**DOI:** 10.1038/s41598-023-42176-0

**Published:** 2023-09-14

**Authors:** Jun-Hyuk Lee, Jang Han Jung, Huiyul Park, Joo Hyun Oh, Sang Bong Ahn, Eileen Laurel Yoon, Dae Won Jun

**Affiliations:** 1 Department of Family Medicine, Nowon Eulji Medical Center, Eulji University, College of Medicine, Daejeon, Republic of Korea; 2 Department of Medicine, Hanyang University School of Medicine, Seoul, Korea; 3 Department of Internal Medicine, Hallym University Dongtan Sacred Heart Hospital, Hallym University, College of Medicine, Chuncheon, Republic of Korea; 4 Department of Family Medicine, Myongji Hospital, Hanyang University College of Medicine, Seoul, Republic of Korea; 5 Department of Internal Medicine, Nowon Eulji Medical Center, Eulji University, College of Medicine, Daejeon, Republic of Korea; 6 Department of Internal Medicine, Hanyang University, College of Medicine, 222 Wangsimni-ro, Seongdong-ru, Seoul, 04763 Republic of Korea; 7 Department of Translational Medicine, Hanyang University Graduate School of Biomedical Science and Engineering, Seoul, Republic of Korea; 8 Department of Internal Medicine, Hanyang University, College of Medicine, 222 Wangsimni-ro, Seongdong-ru, Seoul, 133-791 Republic of Korea

**Keywords:** Non-alcoholic fatty liver disease, Non-alcoholic steatohepatitis, Epidemiology

## Abstract

Non-alcoholic fatty liver disease (NAFLD) is often diagnosed incidentally during medical evaluation for diseases other than liver disease or during health checkups. This study aimed to investigate the awareness, current status, and barriers to the management of NAFLD in the general population. This cross-sectional study used an online survey, which consisted of 3-domain and 18-item questionnaires. The content validity index for each item of the questionnaire was rated above 0.80. Most respondents (72.8%) reported having heard of the term ‘NAFLD’, and a large proportion of the general population (85.7%) recognized the possibility of developing fatty liver without consuming alcohol. Awareness of the terminology of NAFLD and that NAFLD is a disease that needs to be managed is relatively high. However, the knowledge that NAFLD can progress to end-stage liver disease and new cardiovascular diseases is lacking. Only 25.7% of the general population is aware that NAFLD increases the incidence of heart and cerebrovascular diseases. Only 44.7% of those who were incidentally diagnosed during a health check-up were provided with any specific guidance on NAFLD, and more than half (55.3%) were not provided with education or guidance on NAFLD or did not remember it. Only 40.2% of people diagnosed with NAFLD incidentally visited a clinic. The reason for not visiting a clinic for the evaluation of NAFLD varied greatly depending on sex and age group. Only 40.2% of patients visited the clinic after being diagnosed with NAFLD. The reasons for not visiting the clinic after NAFLD diagnosis differed significantly according to sex and age.

## Introduction

Non-alcoholic fatty liver disease (NAFLD) has become the most common cause of chronic liver disease, with a prevalence rate of 24–37.8% globally and 16.1–33.9% in Korea^1–4^. In addition, the number of patients with compensated cirrhosis due to nonalcoholic steatohepatitis (NASH) more than doubled, and that with decompensated cirrhosis due to NASH more than tripled from 1990 to 2017^5^. Accordingly, the economic burden caused by NAFLD was estimated to be $103 billion ($1613 per patient) per year in the United States and €35 billion (€354–1163 per patient) per year in Europe^6^. In addition to the age-standardized mortality rate due to NASH, the socio-economic burden of NAFLD has also dramatically increased worldwide^5,7^. Especially, patients with NAFLD have a 30–60% higher risk of death from malignancy and cardiovascular disease (CVD) compared with people without NAFLD^8–12^.

Awareness and knowledge of NAFLD in the general population are not satisfactory^13–15^, with a large gap between awareness and knowledge. Amandeep et al.^13^ reported that the awareness of NAFLD among adults suspected of having NAFLD was very low (1.5% in 2001–2004 to 3.5% in 2013–2016). Additionally, less than a quarter of patients with high metabolic risk, such as those with overweight/obesity and/or insulin resistance, were aware of NAFLD^14^. More interestingly, one survey of primary care physicians showed that 56% of respondents indicated that NAFLD was related to alcohol consumption.^16^ However, with the recent development of social media and extensive medical check-ups, awareness of the term NAFLD has improved. According to national surveys conducted by the Korean Association for the Study of the Liver^15,17^, the awareness of NAFLD among general Korean adults has significantly increased from 27.9% in 2013 to 72.4% in 2020, which is attributed to the wide availability of media. However, fewer than half of the respondents knew the risk factors associated with nonalcoholic steatohepatitis^17^. Furthermore, the diagnosis of NAFLD remains low, and the treatment cascade that leads to appropriate treatment after diagnosis is inappropriate^15,17^. There is a large gap in the awareness, knowledge, and management of NAFLD in the general population.

A relatively large number of studies on awareness of NAFLD have been reported, but data on the knowledge, current management status, and barriers to management of NAFLD in the general population are limited^18–20^. Two small-scale studies have highlighted that patients with NAFLD face difficulties in making significant behavioral changes despite being aware of their condition.^18,19^ The primary barriers to self-management in patients with NAFLD were insufficient motivation to change behaviors, concurrent medical comorbidities, time and energy constraints, limited weight awareness, consideration of food as a reward or compensation, and social entertainment. A very recent interventional study involving 197 patients with NAFLD revealed that 22% of them were at high-risk health behavior patterns, such as being sedentary, having unhealthy dietary patterns, and having low desire to change their health behaviors, despite a high level of knowledge^20^. In addition, a one-time education intervention aimed at modifying their lifestyle had a positive impact on weight reduction, diet, physical activity, and health-related quality of life at a 6-month follow-up^20^. It is necessary to confirm whether such tendencies are consistent in a large population.

The treatment goal is difficult to achieve if physicians do not understand the barriers and demands of managing NAFLD from the perspective of the general population. Therefore, it is important to understand the awareness and knowledge of NAFLD as well as the barriers and demands for NAFLD in the general population to fill the gap between awareness, knowledge, and the management cascade of NAFLD. This study aimed to assess the current management status of NAFLD and treatment barriers beyond the knowledge and awareness of NAFLD in the general Korean population.

## Results

### Demographics of the study population

Table 1 presents the demographic characteristics of the study population. The mean age was 45.2 years, and the proportion of men was 51.0% among a total of 1000 members of the general population. By age group, 17.7%, 17.6%, 21.9%, 23.3%, and 19.5% of the participants were in their 20 s, 30 s, 40 s, 50 s, and 60 s, respectively. Further, 54.5%, 19.0%, and 26.5% of participants were living in metropolitan areas, urban cities, and rural areas, respectively (Supplementary Fig. 1). The percentages of white-collar, blue-collar, self-employed, homemakers, and unemployed workers were 42.6%, 14.6%, 13.0%, 15.8%, and 14.0%, respectively.

**Table 1:** Demographic information of the study population.

Variables	Number (%)
Men, n (%)	510 (51.0%)
Age, years	45.2 ± 13.7
Age groups, n (%)	
20–29	177 (17.7%)
30–39	176 (17.6%)
40–49	219 (21.9%)
50–59	233 (23.3%)
≥ 60	195 (19.5%)
Body mass index, kg/m^2^	23.3 ± 3.4
Obesity, n (%)	
Normal weight	508 (50.8%)
Overweight	209 (20.9%)
Obese	283 (28.3%)
Residential area, n (%)	
Metropolitan area	545 (54.5%)
Urban cities	190 (19.0%)
Rural area	265 (26.5%)
Monthly household income, n (%)	
Less than one million Korean Won	60 (6.0%)
Between one and three million Korean Won	297 (29.7%)
Between three and five million Korean Won	335 (33.5%)
Over five million Korean Won	308 (30.8%)
Occupation, n (%)	
Corporate office worker	426 (42.6%)
Corporate manual worker	146 (14.6%)
Self-employed worker	130 (13.0%)
Homemaker	158 (15.8%)
Unemployed	140 (14.0%)

### Gap between awareness and knowledge of NAFLD

**Figure 1:**
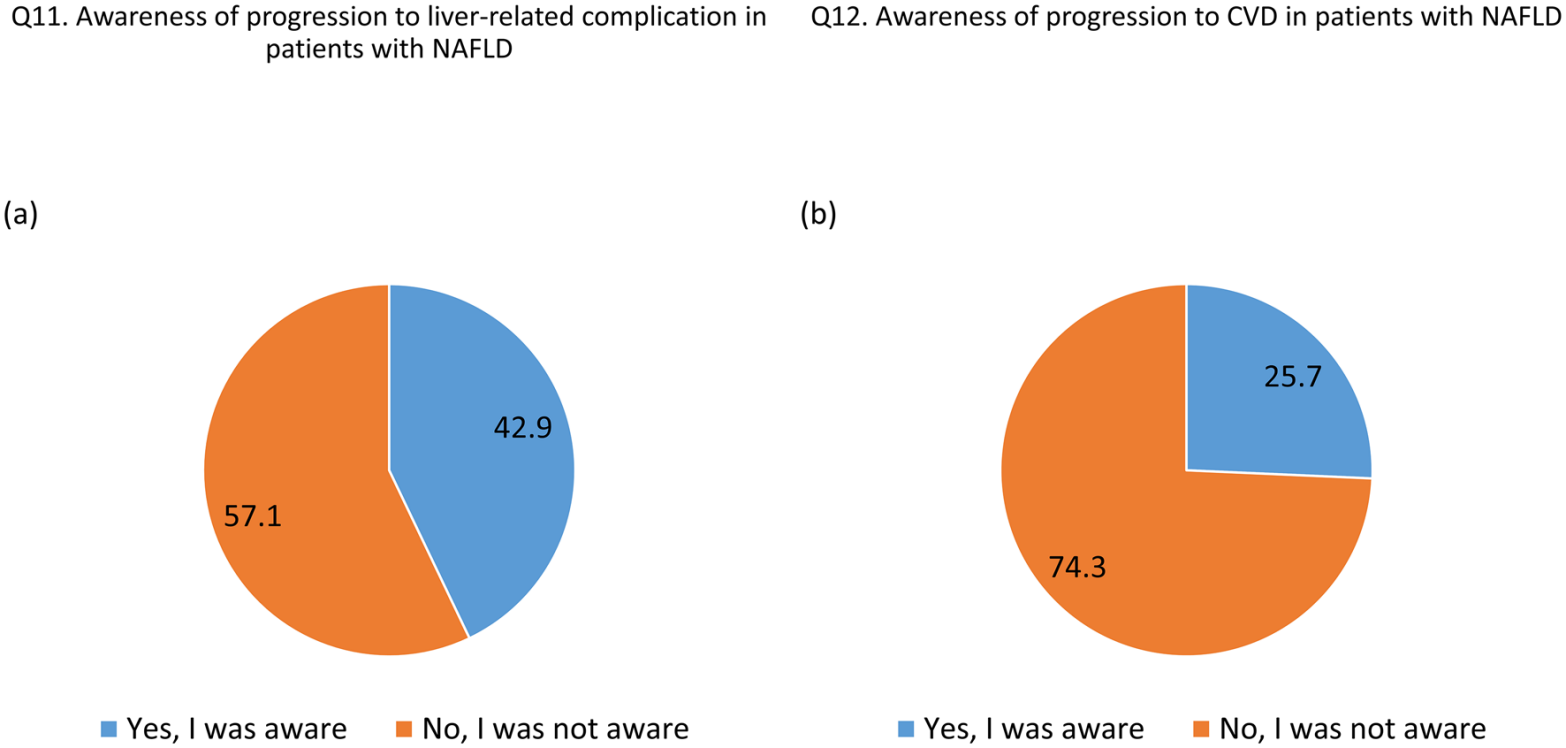
Awareness of possible complications that can progress with persistent NAFLD. Abbreviation: NAFD, non-alcoholic fatty liver disease.

Awareness of NAFLD was relatively high in the general population; however, knowledge of NAFLD was low (Supplementary Table 1). Most respondents (72.8%) reported that they had heard the term 'NAFLD'. Most respondents thought that patients with NAFLD required medical management (82.5%), and a large proportion recognized the possibility of developing fatty liver without alcohol consumption (85.7%). However, only 42.9% of the general population know that NAFLD can progress to liver cirrhosis or hepatocellular carcinoma (Fig. 1A) and only 25.7% of the general population know that NAFLD increases the incidence of heart and cerebrovascular disease (Fig. 1B). Awareness of the terminology for NAFLD and its management is high; however, the knowledge that NAFLD can progress to end-stage liver disease and new cardiovascular diseases is lacking.

### Current management status of NAFLD

**Figure 2:**
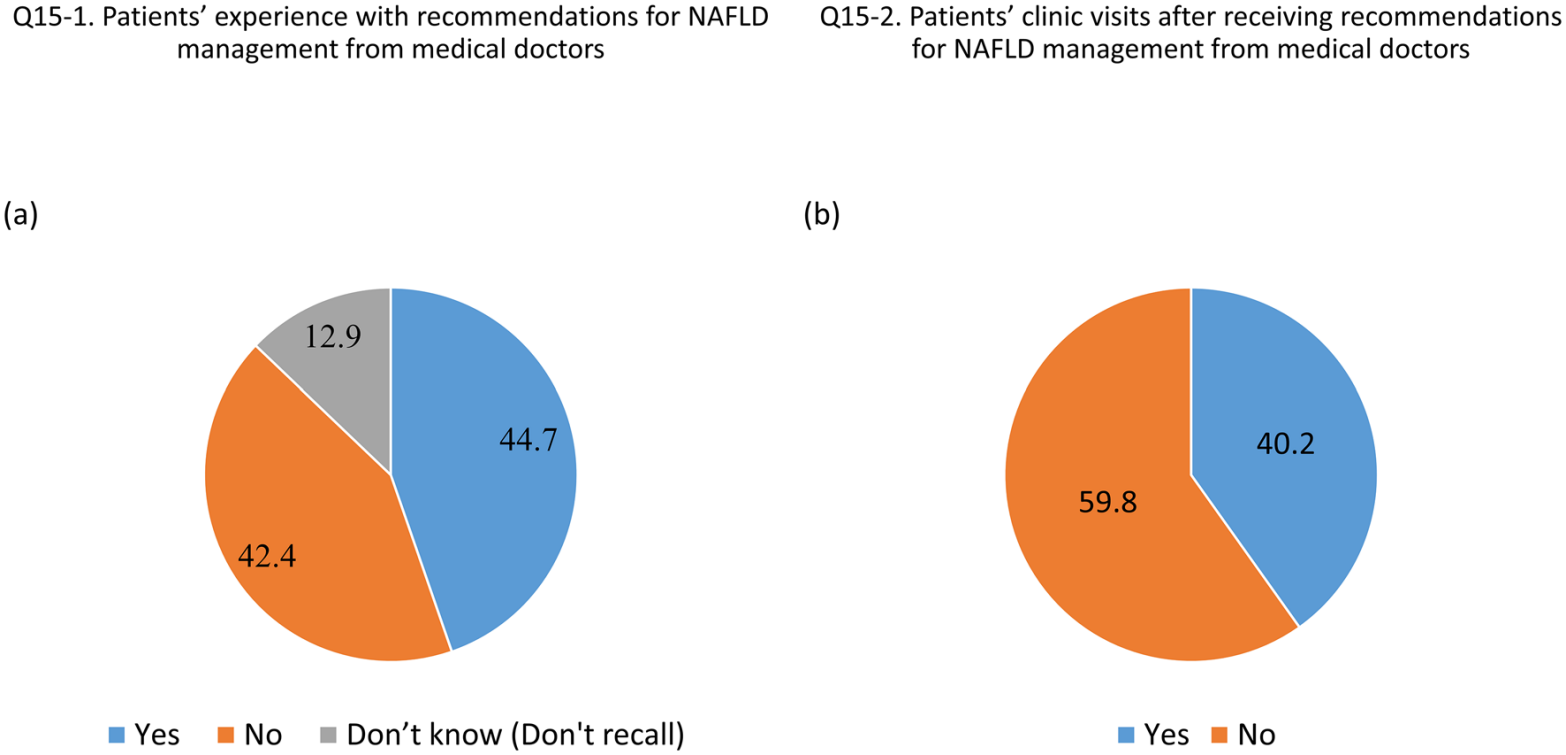
The proportion of patients newly diagnosed with NAFLD who received lifestyle education from healthcare professionals (**A**) and the hospital revisiting rate (**B**). Abbreviation: NAFD, non-alcoholic fatty liver disease.

Supplementary Table 2 shows the responses to questions regarding the current management status and barriers to NAFLD management. Of the 1000 respondents, 13.2% reported that they had been diagnosed with NAFLD during health check-ups or evaluated for diseases other than liver disease. Figure 2A,B show the survey results of medical utilization experiences among the subjects at the time of initial NAFLD diagnosis. Among the patients diagnosed with NAFLD incidentally, only 44.7% were provided with specific guidance for NAFLD by medical staff (Fig. 2A), and only 40.2% of those diagnosed with NAFLD by chance visited a medical clinic for further evaluation and management (Fig. 2B). Meanwhile, 59.3% of patients with NAFLD visited a healthcare facility for NAFLD management among those who received advice on lifestyle modification, while only 24.7% of those diagnosed with NAFLD who were not advised on lifestyle modification visited a medical clinic (Supplementary Fig. 2).

### Barriers to management of NAFLD

**Figure 3:**
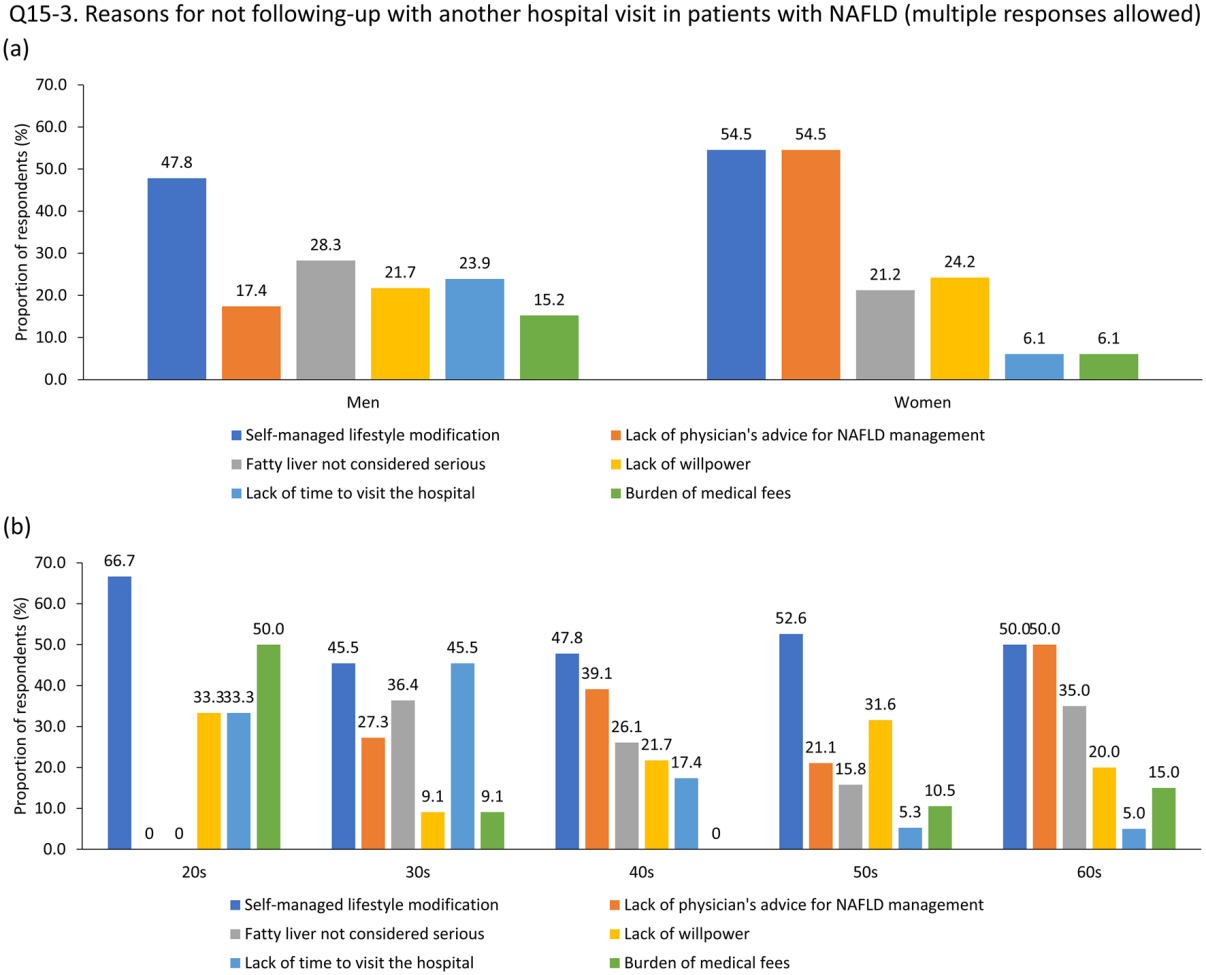
Reasons for not visiting a clinic after being newly diagnosed with NAFLD by gender (**A**) and age group (**B**). Abbreviation: NAFD, non-alcoholic fatty liver disease.

Among the total respondents, as well as subgroups by sex and age groups, the most common reason for not visiting a clinic was ‘I could manage NAFLD by lifestyle modification on my own' (Supplementary Table 2). However, the reasons for not visiting a clinic varied significantly depending on sex and age group (Figs. 3A,B). By sex, 28.3% of men pointed out that they did not consider NAFLD a grave disease, whereas 54.5% of women responded that they had never received an explanation from a doctor that disease management was necessary (Fig. 3A). The reasons for not visiting the clinic after NAFLD diagnosis also differed according to age. Nearly half (50.0%) of participants in their 20 s pointed out the ‘burden of medical fees’, whereas 45.5% of people in their 30 s answered ‘lack of time to visit a clinic’. Individuals in their 40 s (39.1%) and 60 s (50.0%) answered that they had never been informed by a doctor about the necessity of disease management. Meanwhile, people in their 50 s (31.6%) responded that a ‘lack of willpower’ was the main reason for not visiting the clinic (Fig. 3B).

### Unmet needs for management of NAFLD

**Figure 4:**
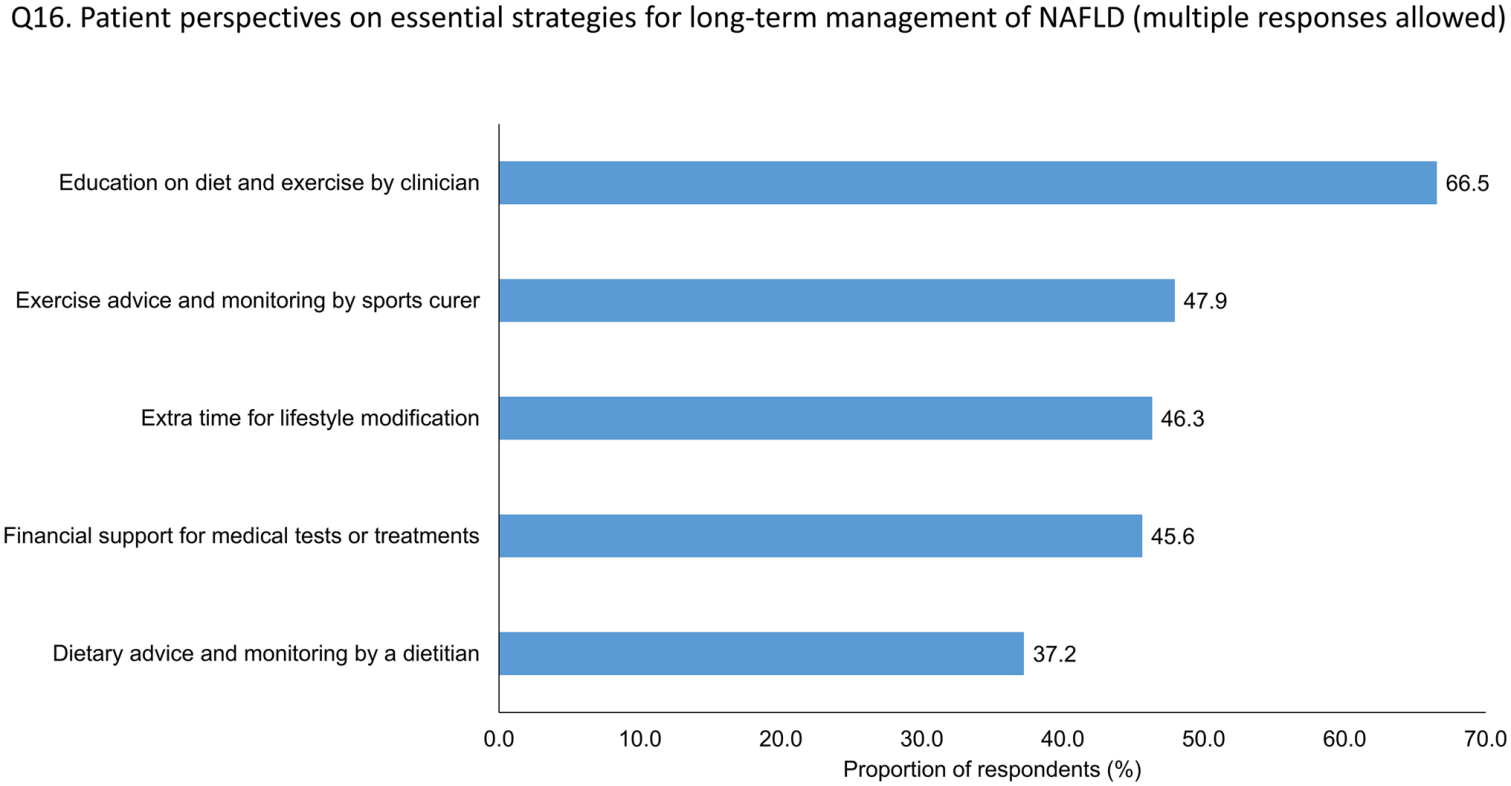
Demands for management of NAFLD in the general population. Abbreviation: NAFD, non-alcoholic fatty liver disease.

Supplementary Table 3 shows the responses to questions concerning unmet needs related to the management of NAFLD in the general population. Of the respondents, 66.5% pointed out that an educational program on lifestyle modifications was provided by a medical doctor (Fig. 4). A considerable proportion of the respondents (60.2%) were willing to participate in the NAFLD management program through mobile applications.

## Discussion

This survey revealed awareness of the terminology for NAFLD; however, the knowledge that NAFLD can progress to end-stage liver disease and incident CVD is lacking in the general population. Only 40.2% of people diagnosed with NAFLD incidentally visited a clinic for further evaluation or management. 28.3 Of the men, 28.3% pointed out that they did not consider NAFLD a grave disease, whereas 54.5% of the women responded that they had never received an explanation from a doctor that disease management was necessary. Participants in their 50 s, accounting for 31.6%, attributed their infrequent clinic visits to a 'lack of willpower'. In contrast, 45.5% of respondents in their 30 s cited a 'lack of time to visit a clinic'. Interestingly, nearly half (50.0%) of participants in their 20 s highlighted the 'burden of medical fees'. Most notably, individuals in their 20 s failed to acknowledge NAFLD as a serious condition and demonstrated a lack of information provided by their physicians (Fig. 3B). This observation suggests a potential shortfall in physician attention towards younger patients, potentially influenced by age bias. Considering the increasing prevalence of NAFLD among young adults^21^, this absence of attention and medical direction could significantly increase their risk of experiencing serious health issues in subsequent years.

The term 'NAFLD' was limited due to its reliance on exclusionary confounder terms and potential perpetuation of stigmatizing language. In 2023, a multi-society consensus was reached among three large pan-national liver associations to redefine the nomenclature 'NAFLD' as 'metabolic-dysfunction associated steatotic liver disease (MASLD)^22^. We anticipate that this new nomenclature, 'MASLD', will help emphasize the association between fatty liver and metabolic dysfunctions to the public, fostering an understanding of the disease's severity and encouraging more proactive lifestyle modifications. However, whether the adoption of this new terminology can effect changes in public perception and behavioral patterns remains to be determined, underscoring the need for further research to understand its impact.

Barriers to NAFLD management vary according to age and sex. Education and management strategies for NAFLD in the general population should be personalized according to age and sex. To the best of our knowledge, this is the first study to analyze the awareness, current practice, and barriers to NAFLD in the general population.

Tincopa et al.^18^ found that while patients with NAFLD were aware of the primary therapy for NAFLD, which is lifestyle modification, they generally lacked knowledge about the condition and were diagnosed incidentally without exhibiting symptoms. The primary facilitators and barriers to lifestyle changes included the presence of social support, competing medical comorbidities, and low motivation. Gu et al.^19^ identified that having basic weight loss knowledge and skills, strong motivation, and attention to NAFLD as key facilitators of weight management. Our study expands on these previous research results by investigating patients’ perspectives on essential strategies for the long-term management of NAFLD. Notably, the proportion of patients who visited a healthcare facility for further evaluation and disease management differed significantly depending on whether they received lifestyle recommendations or guidance from their physicians. Specifically, 59.3% of those who received lifestyle recommendations visited a healthcare facility, while only 24.7% of those who did not receive such recommendations visited a healthcare facility. This implies that healthcare provider education on NAFLD management is a crucial factor in healthcare utilization and motivation for lifestyle modifications.

While almost two-thirds of the participants (58.5%) responded to ‘increasing the amount of exercise’ to prevent and manage NAFLD, only 17% of the respondents identified ‘lack of exercise’ as the most important risk factor for developing NAFLD. There could be a sex difference in the main strategy for weight loss^23–25^. According to Tsai et al.^23^, women were more likely to eat diet foods, join a weight loss program, take prescription diet pills, and follow a special diet, while men were more likely to exercise more and eat less fat. In addition, women with a lower body mass index showed a 40% higher success rate of weight loss than men. Our findings support previous evidence. Hence, different weight reduction programs should be provided for men and women.

Traditionally, written materials are the most commonly preferred method for learning about NAFLD (73%), followed by meeting a healthcare provider (66%), and online learning tools (62%)^14^. With recent advances in digital wearable devices and smartphone applications,^26,27^ our survey results found a different trend compared to previous evidence, as the preference for participating in a smartphone application for the preventive management of NAFLD was found to be higher than the preference for visiting public health centers. This was more pronounced among participants aged 50–60 years than among those aged 20–40 years. However, it should be emphasized that digital therapeutic tools are most effective when used in the context of a weight management intervention or ongoing clinical relationship, rather than being provided alone^28^. As digital therapy becomes more common, it will be an important strategy for the management of patients with NAFLD.

This study had several limitations. First, we only surveyed Koreans. Differences in ethnicity/race, economic status, and healthcare systems between countries can limit the application of our results to other countries. Second, there is the possibility of information bias. As the survey was conducted anonymously online, the accuracy of the responses could not be guaranteed. Third, we did not have access to detailed information on laboratory data, methods for diagnosing NAFLD, physical activity, and dietary patterns. Additionally, we lacked specific data regarding the severity of NAFLD and comorbidities such as diabetes mellitus, hypertension, and dyslipidemia. Finally, only 13.2% of respondents provided feedback on the current management status of NAFLD. It may be difficult to generalize this as representative of the current management status of patients with NAFLD. We also did not include questions regarding whether NAFLD screening was performed in the hospital, the diagnostic method for NAFLD, or whether it was an incidental or supplementary diagnosis. Further surveys targeting only patients with NAFLD are required. Despite these limitations, we first identified healthcare utilization rates after the diagnosis of NAFLD, as well as barriers and demands to manage NAFLD in the general population.

In conclusion, only 40% of Koreans visit healthcare facilities for further evaluation and management after being incidentally diagnosed with NAFLD. This could be due to the insufficient patient education provided by clinicians. Our findings highlight the importance of setting strategies to increase public awareness of NAFLD and establish a NAFLD management strategy in the healthcare system. Further research is needed to verify the cost-effectiveness of applying such strategies to the general population.

## Methods

### Study design and sampling strategy

The survey was conducted online to obtain data from adults over the age of 18 among the online panels owned by an online survey research company, MACROMILL EMBRAIN Co., Ltd. (https://embrain.com/eng/), using quota sampling. The representativeness of the Korean general population was increased by ensuring that sex, age, and residential area had a similar ratio to the population structure of Korea. Between September 2, 2022, and September 16, 2022, 1,207 respondents completed the questionnaire out of 2,105 people who accessed the online survey (response rate 57.3%). We finally included a total of 1000 people in the analysis, after excluding 207 people who provided insufficient responses. The margin of error for a sample of 1000 was ± 3.1%, with a 95% confidence interval.

This study was approved by the Institutional Review Board (IRB) of Hanyang University Hospital (IRB number: 2022-08-01-49). The IRB waived the requirement for informed consent because we used de-identified data provided by the investigating company. All methods were performed following relevant regulations and guidelines.

### Inclusion and exclusion criteria

Panel members registered with the company were eligible to participate in this study. The inclusion criteria were Individuals aged at least 18 years and capable of reading Korean. Additionally, the survey excluded individuals not covered by the National Health Insurance Service or medical doctors. The rest of the panels received e-mails inviting them to participate in the online surveys.

### Domain and contents of questionnaire

Referring to previous survey studies^29–31^, a research group consisting of five physicians (three hepatologists and two primary care physicians specializing in family medicine) developed a 3-domain, 18-item questionnaire on awareness, experience, and demand for NAFLD, as shown in Appendix File 1. The questionnaire was composed of three domains: (1) a 5-item section on demographics and self-perception of obesity; (2) a 9-item section on awareness and knowledge about NAFLD; and (3) a 4-item section on experience, barriers, and demand for NAFLD. In addition, for the 4-item section on experience, barriers, and demand for NAFLD, five additional questions about the management of NAFLD (question numbers 15–1 to 15–5) were assigned to individuals who had been diagnosed with NAFLD.

### Validation of questionnaire

The initial version of the questionnaire was reviewed by seven physicians (four hepatologists, one cardiologist, one endocrinologist, and one neurologist) who agreed to produce a piloted version of the questionnaire. We conducted cognitive interviews with 10 physicians to test whether the respondents’ interpretations matched the intended meaning. This process was repeated four times before the final version of the questionnaire was completed. Content validity was measured by 14 experts, including medical doctors, nutritionists, and methodologists who did not participate in the development of the questionnaire. The content validity index (CVI) for each item was rated above 0.80, and the overall CVI was 0.85. After confirming the content validity, the final version of the questionnaire was read and approved by all developers. Finally, it was translated into English by two native English speakers who were excluded from the study.

### Statistical analysis

All data are expressed as mean ± standard deviation for continuous variables and number (percentage, %) for categorical variables. The chi-squared test was used to compare categorical parameters between men and women or among age groups. Student’s *t*-tests were used to compare continuous parameters between men and women. Analysis of variance was used to compare continuous parameters among age groups. All statistical analyses were performed using the R software (version 4.1.4; R Foundation for Statistical Computing, Vienna, Austria) and SPSS software (version 25.0; IBM Corp., Armonk, New, USA). A *p*-value of less than 0.05 was considered statistical *p* < 0.05.

### Ethical approval

This study was approved by the IRB of Hanyang University Hospital (IRB number:2022-08-01-49). The IRB waived the requirement for informed consent because we used de-identified data provided by the investigating company. All methods were performed following relevant regulations and guidelines.

### Supplementary Information

Supplementary Information 1.

Supplementary Figure 1.

Supplementary Figure 2.

Supplementary Tables.

## Data Availability

All the data were obtained upon the request to the corresponding authors.

## Abbreviations

NAFLD: Non-alcoholic fatty liver disease
NASH: Non-alcoholic steatohepatitis
CVD: Cardiovascular disease
IRB: Institutional review board
