# Supplementary material for: A survey on the awareness, current management, and barriers for non-alcoholic fatty liver disease among the general Korean population
Source: Sci Rep. 2023 Sep 14;13:15205. doi: 10.1038/s41598-023-42176-0 (PMC10502016; doi:10.1038/s41598-023-42176-0)
Supplement: Supplementary file 1 — Supplementary Information 1. [file 41598_2023_42176_MOESM1_ESM.docx]

Questionnaire for the General Public

In nonalcoholic fatty liver disease, chronic inflammation and fibrosis in the liver caused by the accumulation of excessive fat increases the mortality from liver diseases. In addition, as a risk factor for cardiovascular diseases such as angina, myocardial infarction, and stroke, it also increases mortality from cardiovascular diseases. The purpose of this questionnaire is to understand the level of general public awareness in order to identify gaps that will inform planning for promotion and education. In addition, identifying limitations of access to treatment for nonalcoholic fatty liver disease will enable us to establish a plan for improvement. All collected data, including personal information will be kept confidential in accordance with the Personal Information Protection Act. Data analysis will be carried out utilizing the integrated opinion of all study participants, and will not be based on your responses alone.

1. What is your height in centimeters?

_________________cm

2. What is your weight in kilograms?

_________________kg

3. How would you describe your body shape?

1. Thin
2. Average
3. Overweight
4. Obese

4. What is your household income?

1. Less than 1 million won per month
2. Between 1 – 3 million won per month
3. Between 3 – 5 million won per month
4. More than 5 million won per month

5. What is your occupation?

1. Corporate office worker
2. Corporate manual worker
3. Self-employed worker
4. Homemaker
5. Unemployed

The following are questions regarding the perception of nonalcoholic fatty liver disease.

6. Do you think you can develop fatty liver without drinking alcohol?

1. Yes
2. No
3. Don’t know

7. Have you ever heard of the term nonalcoholic fatty liver disease?

1. Never heard before
2. At the hospital
3. On TV
4. On the radio
5. In the newspaper
6. On YouTube
7. On the internet web portal
8. During a health checkup

If ① is selected in question 5, provide definition: Nonalcoholic fatty liver disease is a disease in which fatty liver is present without significant factors such as drinking, drugs, viral hepatitis, etc.

8. How do you rate your understanding regarding nonalcoholic fatty liver disease?

1. Very high level of understanding (5points)
2. High level of understanding (4points)
3. Average level of understanding (3points)
4. Low level of understanding (2points)
5. No understanding (1point)

9. What percentage of the Korean population do you think has nonalcoholic fatty liver disease?

1. Less than 10%
2. Between 10 – 20%
3. Between 20 – 30%
4. Between 30 – 40%
5. Between 40 – 50%
6. More than 50%

10. Do you think nonalcoholic fatty liver disease is a disease that needs to be treated at a hospital?

1. Strongly agree (5points)
2. Agree (4points)
3. Don’t know (3points)
4. Disagree (2points)
5. Strongly disagree (1point)

11. If nonalcoholic fatty liver disease persists, it could progress to chronic liver inflammation/liver cirrhosis/liver cancer. Were you aware of this?

1. Yes, I was aware
2. No, I was not aware

12. If nonalcoholic fatty liver disease persists, it could progress to angina/myocardial infarction/cerebral stroke (palsy). Were you aware of this?

1. Yes, I was aware.
2. No, I was not aware.

13. What do you think are the most common symptoms in patients with nonalcoholic fatty liver disease?

1. Mostly asymptomatic.
2. Jaundiced appearance.
3. Abdominal pain.
4. Edema.
5. Nausea and vomiting.

14. In order of greatest risk factor to smallest risk factor, select from the following risk factors that can cause nonalcoholic fatty liver disease (1^st^, 2^nd^, 3^rd^, 4^th^, 5^th^, 6^th^).

1. Aging
2. Genetic factor
3. Drinking
4. Diabetes
5. Obesity
6. Lack of exercise

The following are questions regarding your experience with nonalcoholic fatty liver disease.

15. Have you ever been told that you have nonalcoholic fatty liver disease?

1. Yes (Go to question 15-1)
2. No (Go to question 16)

15-1. At the hospital, have you ever been recommended lifestyle modification for the treatment of nonalcoholic fatty liver disease?

1. Yes
2. No
3. Don’t know (Don’t recall)

15-2. After receiving diagnosis of nonalcoholic fatty liver disease, did you visit the hospital for further tests and management of the disease?

1. Yes (Go to question 15-4)
2. No (Go to question 15-3)

15-3. What was your reason for not following-up with another hospital visit?

1. Did not consider fatty liver a grave disease
2. Thought I could manage the disease by taking on lifestyle modification on my own (weight management, exercise management, etc.)
3. Lack of time to visit the hospital
4. Burden of medical fees
5. Lack of willpower
6. Never been told from my physician that I need disease management

15-4. If there is something you are doing on a regular basis for the prevention or management of nonalcoholic fatty liver disease, please select them in order of priority. (Multiple responses allowed)

1. I am not doing anything in particular in management of my nonalcoholic fatty liver disease.
2. Liver supplements sold at drug stores or though home shopping
3. Supplements for hyperlipidemia sold at drug stores or through home shopping
4. Liver medication prescribed at the hospital
5. Hyperlipidemia medication prescribed at the hospital
6. Diet medication
7. Reduction in calorie intake
8. Increase in the amount of exercise
9. Weight loss

15-5. What is the greatest obstacle to modifying your lifestyle and maintaining that change for management of your nonalcoholic fatty liver disease?

1. I am not certain of the reason for lifestyle modification
2. Lack of time
3. Lack of willpower
4. Lack of information
5. Unable to receive continuous feedback

16. What do you think is most essential to effective management of nonalcoholic fatty liver disease in the long-term? (Multiple responses allowed)

1. Time set aside for lifestyle modification
2. Health with medical bills
3. Dietary advice and periodic management provided by a dietician
4. Advice on how to exercise and periodic management provided by a sports curer
5. Education regarding appropriate diet and exercise provided by a clinician

17. If there is a cellphone application for the prevention or management of nonalcoholic fatty liver disease, would you be willing to participate?

1. Willing to actively participate (5points)
2. Willing to participate (4points)
3. Neutral (3points)
4. Little interest in participating (2points)
5. No interest in participating (1point)

18. If there is a public health center visiting program for the prevention or management of nonalcoholic fatty liver disease, would you be willing to participate?

1. Willing to actively participate (5points)
2. Willing to participate (4points)
3. Neutral (3points)
4. Little interest in participating (2points)
5. No interest in participating (1point)
