# Supplementary figures and images for: A survey on the awareness, current management, and barriers for non-alcoholic fatty liver disease among the general Korean population
Source: Sci Rep. 2023 Sep 14;13:15205. doi: 10.1038/s41598-023-42176-0 (PMC10502016; doi:10.1038/s41598-023-42176-0)

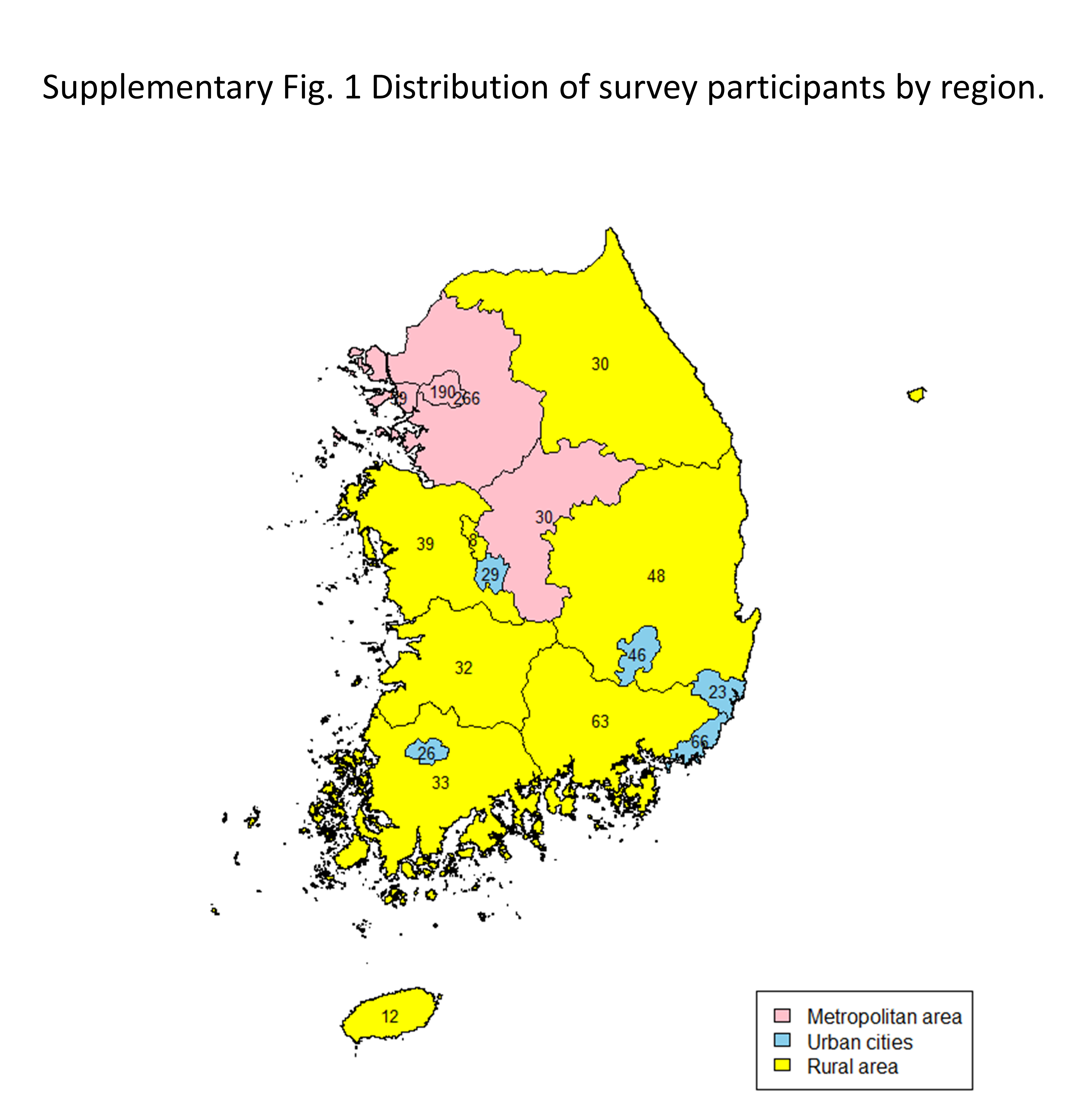

Supplement: Supplementary file 2 — Supplementary Figure 1. [file 41598_2023_42176_MOESM2_ESM.tif]

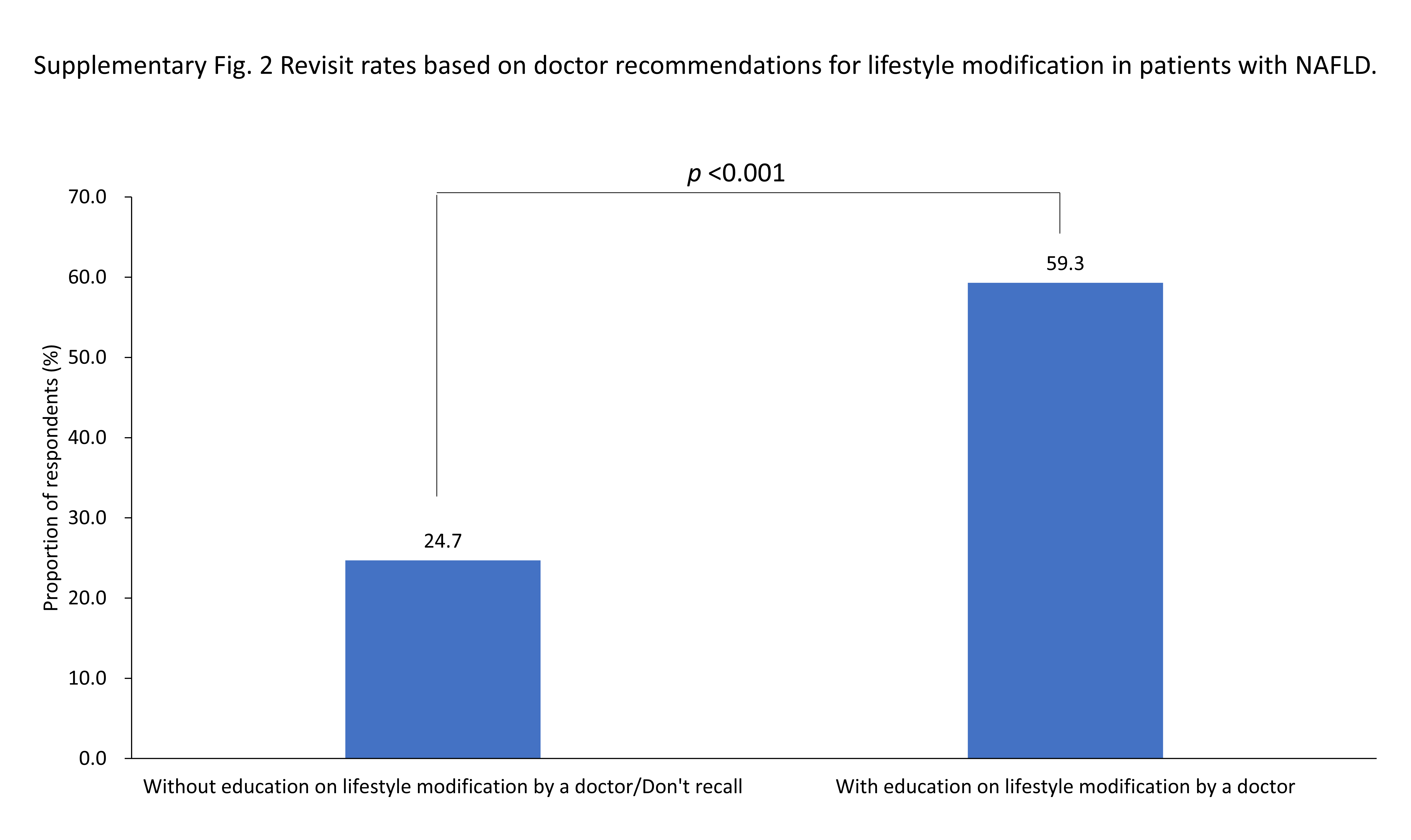

Supplement: Supplementary file 3 — Supplementary Figure 2. [file 41598_2023_42176_MOESM3_ESM.tif]
