# Supplementary material for: A survey on the awareness, current management, and barriers for non-alcoholic fatty liver disease among the general Korean population
Source: Sci Rep. 2023 Sep 14;13:15205. doi: 10.1038/s41598-023-42176-0 (PMC10502016; doi:10.1038/s41598-023-42176-0)
Supplement: Supplementary file 4 — Supplementary Tables. [file 41598_2023_42176_MOESM4_ESM.doc]

Supplementary Tables

Title: A survey on the awareness, current management, and barriers for non-alcoholic fatty liver disease among the general Korean population

Authors: Jun-Hyuk Lee^1,2*^, Jang Han Jung^3*^, Huiyul Park^4^, Joo Hyun Oh^5^, Sang Bong Ahn^5^, Eileen Laurel Yoon^6,7†^, and Dae Won Jun^6,7†^

^1^Department of Family Medicine, Nowon Eulji Medical Center, Eulji University, College of Medicine,

^2^Department of Medicine, Hanyang University School of Medicine,

^3^Department of Internal Medicine, Hallym University Dongtan Sacred Heart Hospital, Hallym University, College of Medicine,

^4^Department of Family Medicine, Myongji Hospital, Hanyang University College of Medicine,

^5^Department of Internal Medicine, Nowon Eulji Medical Center, Eulji University, College of Medicine,

^6^Department of Internal Medicine, Hanyang University, College of Medicine,

^7^Department of Translational Medicine, Hanyang University Graduate School of Biomedical Science and Engineering, Seoul, Korea

^*^These authors contributed equally to this work as first authors.

^†^These authors contributed equally to this work as co-corresponding authors.

*Corresponding authors:

Dae Won Jun, MD, PhD

Department of Internal Medicine, Hanyang University College of Medicine

222 Wangsimni-ro, Seongdong-gu, Seoul 04763, Korea

E-mail: [noshin@hanyang.ac.kr](mailto:noshin@hanyang.ac.kr)

Phone: +82 2 2290 8338; Fax: +82 2 972 0068

or

Eileen L. Yoon

222 Wangsimni-ro, Seongdong-gu, Seoul, 133-791, Department of Internal Medicine

Hanyang University College of Medicine, Republic of Korea

Tel: +82 2 2290 8338, Fax: +82 2 972 0068, E-mail: [mseileen80@hanyang.ac.kr](mailto:mseileen80@hanyang.ac.kr)

Supplementary Table 1. Gap between awareness and knowledge of NAFLD

| Question | Number (%) |
| --- | --- |
| Q6. Do you think you can develop fatty liver without drinking alcohol? |  |
| Yes | 857 (85.7%) |
| No | 20 (2.0%) |
| Don’t know | 123 (12.3%) |
| Q7. Have you ever heard of the term nonalcoholic fatty liver disease? (Multiple responses allowed) |  |
| Never heard before | 272 (27.2%) |
| At the hospital | 248 (24.8%) |
| On TV | 430 (43.0%) |
| On the radio | 56 (5.6%) |
| In the newspaper | 116 (11.6%) |
| On YouTube | 188 (18.8%) |
| On the internet web portal | 326 (32.6%) |
| During a health checkup | 225 (22.5%) |
| Q8. How do you rate your understanding regarding nonalcoholic fatty liver disease? |  |
| Very high level of understanding (5points) | 22 (2.2%) |
| High level of understanding (4points) | 94 (9.4%) |
| Average level of understanding (3points) | 316 (31.6%) |
| Low level of understanding (2points) | 276 (27.6%) |
| No understanding (1point) | 292 (29.2%) |
| Q9. What percentage of the Korean population do you think has nonalcoholic fatty liver disease? |  |
| Less than 10% | 274 (27.4%) |
| Between 10–20% | 386 (38.6%) |
| Between 20–30% | 237 (23.7%) |
| Between 30–40% | 70 (7.0%) |
| Between 40–50% | 15 (1.5%) |
| More than 50% | 18 (1.8%) |
| Q10. Do you think nonalcoholic fatty liver disease is a disease that needs to be treated at a hospital? |  |
| Strongly agree (5points) | 382 (38.2%) |
| Agree (4points) | 443 (44.3%) |
| Don’t know (3points) | 134 (13.4%) |
| Disagree (2points) | 37 (3.7%) |
| Strongly disagree (1point) | 4 (0.4%) |
| Q11. If nonalcoholic fatty liver disease persists, it could progress to chronic liver inflammation/liver cirrhosis/liver cancer. Were you aware of this? |  |
| Yes, I was aware | 429 (42.9%) |
| No, I was not aware | 571 (57.1%) |
| Q12. If nonalcoholic fatty liver disease persists, it could progress to angina/myocardial infarction/cerebral stroke (palsy). Were you aware of this? |  |
| Yes, I was aware | 257 (25.7%) |
| No, I was not aware | 743 (74.3%) |
| Q13. What do you think are the most common symptoms in patients with nonalcoholic fatty liver disease? (Multiple responses allowed) |  |
| Mostly asymptomatic. | 521 (52.1%) |
| Jaundiced appearance. | 349 (34.9%) |
| Abdominal pain. | 148 (14.8%) |
| Edema. | 183 (18.3%) |
| Nausea and vomiting. | 125 (12.5%) |
| Q14. In order of greatest risk factor to smallest risk factor, select from the following risk factors that can cause nonalcoholic fatty liver disease (1st, 2nd, 3rd, 4th, 5th, 6th). |  |
| Aging | 40 (4.0%) |
| Genetic factor | 201 (20.1%) |
| Drinking | 128 (12.8%) |
| Diabetes | 380 (38.0%) |
| Obesity | 380 (38.0%) |
| Lack of exercise | 170 (17.0%) |

Abbreviation: NAFLD, nonalcoholic fatty liver disease.

Supplementary Table 2. Current management status of and barriers to NAFLD

| Question | Number (%) |
| --- | --- |
| Q15. Have you ever been told that you have nonalcoholic fatty liver disease? |  |
| Yes (Go to question 15-1) | 132 (13.2%) |
| No (Go to question 16) | 868 (86.8%) |
| Q15-1. At the hospital, have you ever been recommended lifestyle modification for the treatment of nonalcoholic fatty liver disease? |  |
| Yes | 59 (44.7%) |
| No | 56 (42.4%) |
| Don’t know (Don’t recall) | 17 (12.9%) |
| Q15-2. After receiving diagnosis of nonalcoholic fatty liver disease, did you visit the hospital for further tests and management of the disease? |  |
| Yes (Go to question 15-4) | 53 (40.2%) |
| No (Go to question 15-3) | 79 (59.8%) |
| Q15-3. What was your reason for not following-up with another hospital visit? (Multiple responses allowed) |  |
| Did not consider fatty liver a grave disease | 20 (25.3%) |
| Thought I could manage the disease by taking on lifestyle modification on my own (weight management, exercise management, etc.) | 40 (50.6%) |
| Lack of time to visit the hospital | 13 (16.5%) |
| Burden of medical fees | 9 (11.4%) |
| Lack of willpower | 18 (22.8%) |
| Never been told from my physician that I need disease management | 26 (32.9%) |
| Q15-4. If there is something you are doing on a regular basis for the prevention or management of nonalcoholic fatty liver disease, please select them in order of priority. (Multiple responses allowed) |  |
| I am not doing anything in particular in management of my nonalcoholic fatty liver disease. | 2 (3.8%) |
| Liver supplements sold at drug stores or though home shopping | 10 (18.9%) |
| Supplements for hyperlipidemia sold at drug stores or through home shopping | 7 (13.2%) |
| Liver medication prescribed at the hospital | 14 (26.4%) |
| Hyperlipidemia medication prescribed at the hospital | 24 (45.3%) |
| Diet medication | 6 (11.3%) |
| Reduction in calorie intake | 22 (41.5%) |
| Increase in the amount of exercise | 31 (58.5%) |
| Weight loss | 29 (54.7%) |
| Q15-5. What is the greatest obstacle to modifying your lifestyle and maintaining that change for management of your nonalcoholic fatty liver disease? |  |
| I am not certain of the reason for lifestyle modification | 10 (7.6%) |
| Lack of time | 5 (3.8%) |
| Lack of willpower | 71 (53.8%) |
| Lack of information | 17 (12.9%) |
| Unable to receive continuous feedback | 29 (22.0%) |

Abbreviation: NAFLD, nonalcoholic fatty liver disease.

Supplementary Table 3. Unmet demands for management of NAFLD

| Question | Number (%) |
| --- | --- |
| Q16. What do you think is most essential to effective management of nonalcoholic fatty liver disease in the long-term? (Multiple responses allowed) |  |
| Time set aside for lifestyle modification | 463 (46.3%) |
| Health with medical bills | 456 (45.6%) |
| Dietary advice and periodic management provided by a dietician | 372 (37.2%) |
| Advice on how to exercise and periodic management provided by a sports curer | 479 (47.9%) |
| Education regarding appropriate diet and exercise provided by a clinician | 665 (66.5%) |
| Q17. If there is a cellphone application for the prevention or management of nonalcoholic fatty liver disease, would you be willing to participate? |  |
| Willing to actively participate (5points) | 150 (15.0%) |
| Willing to participate (4points) | 452 (45.2%) |
| Neutral (3points) | 294 (29.4%) |
| Little interest in participating (2points) | 85 (8.5%) |
| No interest in participating (1point) | 19 (1.9%) |
| Q18. If there is a public health center visiting program for the prevention or management of nonalcoholic fatty liver disease, would you be willing to participate? |  |
| Willing to actively participate (5points) | 102 (10.2%) |
| Willing to participate (4points) | 371 (37.1%) |
| Neutral (3points) | 362 (36.2%) |
| Little interest in participating (2points) | 141 (14.1%) |
| No interest in participating (1point) | 24 (2.4%) |

Abbreviation: NAFLD, nonalcoholic fatty liver disease.
